# Overstimulation of the inhibitory nervous system plays a role in the pathogenesis of neuromuscular and neurological diseases: a novel hypothesis

**DOI:** 10.12688/f1000research.8774.2

**Published:** 2016-08-19

**Authors:** Bert Tuk

**Affiliations:** 1 Leiden Academic Center for Drug Research (LACDR), Leiden University, Leiden, 2333 CC, Netherlands; 2 Ry Pharma, Hofstraat 1, Willemstad, 4797 AC, Netherlands

**Keywords:** Neuromuscular disease, neurodegeneration, ALS, FTD, Alzheimer’s disease, Parkinson’s disease, Huntington’s disease, Primary Lateral Sclerosis

## Abstract

Based upon a thorough review of published clinical observations regarding the inhibitory system, I hypothesize that this system may play a key role in the pathogenesis of a variety of neuromuscular and neurological diseases. Specifically, excitatory overstimulation, which is commonly reported in neuromuscular and neurological diseases, may be a homeostatic response to inhibitory overstimulation. Involvement of the inhibitory system in disease pathogenesis is highly relevant, given that most approaches currently being developed for treating neuromuscular and neurological diseases focus on reducing excitatory activity rather than reducing inhibitory activity.

## The clinical manifestations of neuromuscular and neurological diseases have high overlap

The pathogenesis of most neuromuscular and neurological diseases is poorly understood, despite their devastating impact on quality of life and the fact that they were first described more than a century ago. Clinically, neuromuscular diseases manifest as progressive muscle weakness together with a general set of motor symptoms, including speech-related difficulties, impaired mobility, and reduced fine motor skills ^1^. In contrast, neurological diseases manifest primarily as a progressive decline in cognitive function. Interestingly, the clinical manifestations of neuromuscular and neurological diseases also overlap; this overlap is summarized in Table 1 for primary lateral sclerosis (PLS), amyotrophic lateral sclerosis (ALS), ALS with frontotemporal dementia (ALS-FTD), FTD with ALS (FTD-ALS), FTD, Alzheimer’s disease, Parkinson’s disease, and Huntington’s disease ^1– 29^. The clinical features shared between the neuromuscular disease ALS and the neurological disease FTD exemplify this overlap, as late-stage ALS can lead to the manifestation of FTD; conversely, FTD can progress to ALS, leading to the manifestation of FTD-ALS ^2– 8^.

**Table 1.** Overview of the clinical manifestations in eight progressive neuromuscular and neurological diseases ^1– 29^.

Clinical manifestation	PLS	ALS	ALS-FTD	FTD-ALS	FTD	Alzheimer’s disease	Parkinson’s disease	Huntington’s disease
Elevated glutamate CSF levels	√	√	√	√	√	√	√	√
Elevated epileptic activity	–	–	–	–	–	–	–	–
Dysphagia	√	√	√	√	√	√	√	√
Dysarthria	√	√	√	√	√	√	√	√
Eye movement difficulties	√	√	√	√	√	√	√	√
Bladder dysfunction	√	√	√	√	√	√	√	√
Gastrointestinal dysfunction	√	√	√	√	√	√	√	√
Cognitive dysfunction	–	√	√	√	√	√	√	√
Rest tremor	–	–	–	–	–	–	√	–
Respiratory depression	√	√	√	√	–	√	√	√
Coordination difficulties	√	√	√	√	–	√	√	√
Impaired muscle function	√	√	√	√	–	√	√	√
Severe muscle wasting	–	√	√	√	–	–	–	–

√, present; –, absent in most patients PLS, primary lateral sclerosis; ALS, amyotrophic lateral sclerosis; FTD, frontotemporal dementia; CSF, cerebrospinal fluid

## Elevated glutamate levels are involved in the pathogenesis of both neuromuscular and neurological diseases

A key observation gleaned from analyzing Table 1 is the finding that glutamate levels are increased in the cerebrospinal fluid (CSF) of patients in all eight diseases ^22– 29^. Glutamatergic (i.e., excitatory) overstimulation induces excitotoxicity in cultured neurons and is believed to be an important factor in the pathogenesis of both neuromuscular and neurological diseases ^22– 29^. Glutamate-induced excitotoxicity can result in the decay of neuronal pathways that innervate muscles and other physiological systems ^22– 29^. This decay gives rise to the loss of physiological function and is considered to lead to the clinical manifestations that present with both neuromuscular disease and neurological disease ^22– 29^. I hypothesize that these increased glutamate levels are actually a homeostatic response to an overstimulated inhibitory nervous system. This novel hypothesis is based upon the observation that the clinical findings in neuromuscular and neurological diseases can be explained by inhibitory activity, as discussed below.

## Despite increased glutamate levels, patients with neuromuscular and neurological diseases do not have increased epileptic activity

Since they were first diagnosed more than a century ago, the clinical manifestations of neuromuscular and neurologic diseases have been well described. Strikingly, however, the consequences of one key clinical feature of these diseases—the absence of an elevated risk of seizure activity—have been largely overlooked.

This is exemplified for ALS in which a broad, detailed retrospective study of the medical records of 657 ALS patients revealed that none of the patients presented with epilepsy as a co-morbid condition ^9^. Moreover, a thorough search of PubMed for articles published from 1966 through 2016 using the key words “seizure” or “epilepsy” in combination with “amyotrophic lateral sclerosis” or “ALS” confirms the striking absence of epilepsy and/or seizures in ALS patients. This finding is consistent with the absence of seizures and/or epilepsy in review articles describing the clinical manifestation of ALS ^2– 6^.

A key observation that makes the absence of seizure activity in ALS even more remarkable is increased glutamate levels in the cerebrospinal fluid (CSF) of patients with ALS; on average, glutamate levels in the CSF of ALS patients are increased by 100%, and some ALS patients can have an increase of up to 800% ^23^. Importantly, increased glutamate levels are generally associated with epileptic seizures ^30, 31^. Thus, given the increased glutamate levels typically measured in the CSF of ALS patients, one would logically expect that the prevalence of epilepsy in ALS patients should be elevated relative to the general population. However, despite this expectation, epileptic seizures are simply not reported among ALS patients.

Strikingly, in addition to ALS, none of the other seven diseases listed in Table 1 typically present with an increased risk of epileptic seizures, either ^2– 15^, even though all eight diseases present with elevated glutamate levels in the CSF ^22– 29^. With respect to Alzheimer’s disease, patients in the early stages of the disease occasionally develop seizures ^14, 32^; however, this seizure activity decreases as the disease progresses from the early stages to more advanced stages ^14, 32, 33^.

## Despite elevated glutamate levels, muscles in neuromuscular and neurological patients are inhibited

A second key observation is that neuromuscular and neurological diseases have an inhibitory effect on muscle function, rather than being excitatory. The diseases listed in Table 1 are characterized by muscle inhibition, even though glutamate—which, as discussed above, is generally increased in these diseases—is the major neurotransmitter that drives muscle activation by increasing the firing rate of motor neurons. Remarkably, however, despite having increased levels of glutamate in the CSF, patients with neuromuscular and neurological diseases do not have increased muscle activation. This is exemplified most clearly by ALS, a disease with highly elevated glutamate levels ^22, 23^ and complete muscle inhibition in the end stages. Although fasciculation and/or cramps can be observed in ALS patients ^2– 4^, these features occur in debilitated muscles as they progress from a fully functional state toward a fully inhibited state.

One possible explanation for these seemingly contradictory findings is a second system that exerts strong anticonvulsive activity in both neuromuscular and neurological diseases. Importantly, such a system should be as widespread throughout the nervous system as the glutamatergic system, and the inhibitory system fulfills these requirements. Specifically, the inhibitory (GABA) system *i*) functions to oppose the glutamatergic excitatory neurotransmitter system, *ii*) inhibits muscle activity by reducing the firing rate of motor neurons, and *iii*) exerts strong anticonvulsive activity ^26, 30, 31^.

## The clinical features of neuromuscular and neurological diseases can be induced by increasing inhibitory activity

A third key observation is that the clinical manifestations of neuromuscular and neurological diseases can be induced using interventions that increase GABAergic (i.e., inhibitory) activity ( Table 2). For example, activating the GABAergic inhibitory system using benzodiazepines can render healthy muscles dysfunctional ^34, 35^. In addition, fatal respiratory depression can be induced by administering an overdose of the GABAergic benzodiazepine midazolam ^36^. Chronically stimulating the inhibitory system can cause chronic muscle disuse that can lead to muscle atrophy ^37^. Moreover, ingestion of alcohol (another GABAergic inhibitory compound ^38^) impedes coordination and causes slurred speech (dysarthria), which are features of neuromuscular and neurological diseases. In cats, dysphagia (difficulty swallowing) can be either induced or reversed using GABA agonists or GABA antagonists, respectively ^39^. Dysphagia has also been reported in humans following the administration of either benzodiazepines ^40– 42^ or alcohol ^43^. Administration of benzodiazepines reduces voluntary saccadic eye movement function ^44^ and increases EEG beta-wave activity ^44^, clinical manifestations that also occur in neuromuscular and neurological diseases ^18, 45, 46^. Increased GABAergic inhibitory activity can also cause bladder ^47, 48^ and gastrointestinal dysfunction ^49, 50^, both of which can manifest in neuromuscular and neurological diseases ^19– 21^. Interestingly, Bravo *et al.* reported that chronically feeding mice the lactic acid bacterium *L. rhamnosus* increases expression of GABA receptors, suggesting a link between GABAergic activity and CNS disorders ^51^. Strikingly, GABAergic activity can also explain the overlapping clinical manifestations between Alzheimer’s disease and alcohol-related dementia ^52^, and it can explain the increase in dementia-like symptoms observed after the administration of the benzodiazepine diazepam ^53^. Inhibitory activity can also explain neuromuscular and neurological disease predisposition in the elderly, as the sensitivity to GABA inhibitory activity is known to increase with age ^54^. Finally, GABAergic activity has been implicated in cognitive dysfunction ^55– 57^, which is a hallmark feature of neurological diseases and is often observed in late-stage neuromuscular disease ^2– 7^. Taken together, these findings support the notion that the clinical features associated with neuromuscular and neurological diseases can be induced by activating the inhibitory system.

**Table 2.** The clinical manifestations of neuromuscular and neurological diseases can be induced by administering compounds that increase inhibitory activity ^31, 34– 50, 52– 57^.

Clinical manifestation	Role of the inhibitory system
Dysphagia	GABAergic compound administration leads to dysphagia that can be reversed by the administration of GABA antagonists
Dysarthria	GABAergic alcohol ingestion can lead to dysarthria
Eye movement dysfunction	GABAergic benzodiazepine administration can lead to eye movement dysfunction
Bladder dysfunction	GABAergic activity can lead to bladder dysfunction
Bowel dysfunction	GABAergic activity can lead to bowel dysfunction
Cognitive dysfunction	GABAergic benzodiazepine administration can lead to increases in dementia scores
Dementia	Long-term GABAergic alcohol ingestion can lead to alcohol-related dementia
Respiratory depression	GABAergic benzodiazepine administration can lead to respiratory depression
Coordination difficulties	GABAergic alcohol ingestion can lead to coordination difficulties
Muscle dysfunction	GABAergic benzodiazepine administration can lead to muscle dysfunction
Muscle blockade	GABAergic benzodiazepine administration can lead to muscle blockade even causing respiratory depression-related fatalities
Muscle atrophy	GABAergic benzodiazepine administration can lead to muscle blockades that leads to muscle disuse that is associated with muscle atrophy
Muscle wasting	GABAergic benzodiazepine administration can lead to muscle blockades that leads to muscle disuse that is associated with loss of muscle mass
ALS mortality	GABAergic activity can account for the faster disease progression observed in clinical trials where ALS patients are treated with GABAergic compounds

## Modulating inhibitory activity can explain the progression of ALS in clinical trials

In addition to mimicking the majority of clinical manifestations observed in neuromuscular and neurological diseases, GABAergic activity can also explain the more rapid disease progression of ALS reported in clinical trials in which patients received GABAergic compounds. For example, in two trials gabapentin increased the rate of disease progression in patients with ALS ^58^. A similar effect was reported in patients with ALS who received the GABAergic compound topiramate ^59^. GABAergic action can also explain the more rapid disease progression of ALS in clinical trials in which patients received the antibiotic minocycline ^60^, which has GABAergic activity ^61^. Finally, GABAergic involvement can explain the observed efficacy of the taurine conjugate form of ursodeoxycholic acid (UDCA) in ALS patients ^62^, as UDCA inhibits GABAergic action ^63^.

## Neuromuscular and neurological manifestations can be attributed to simple inhibition and/or recurrent inhibition

A fourth key observation is that the clinical manifestations associated with neuromuscular and neurological diseases can be attributed to the activity of either simple inhibition (SI) or recurrent inhibition (RI) pathways. Specifically, I postulate that differences between muscles under the control of SI and/or RI underlie the important—yet poorly understood—manifestations of neuromuscular and neurological diseases.

**Figure 1.**
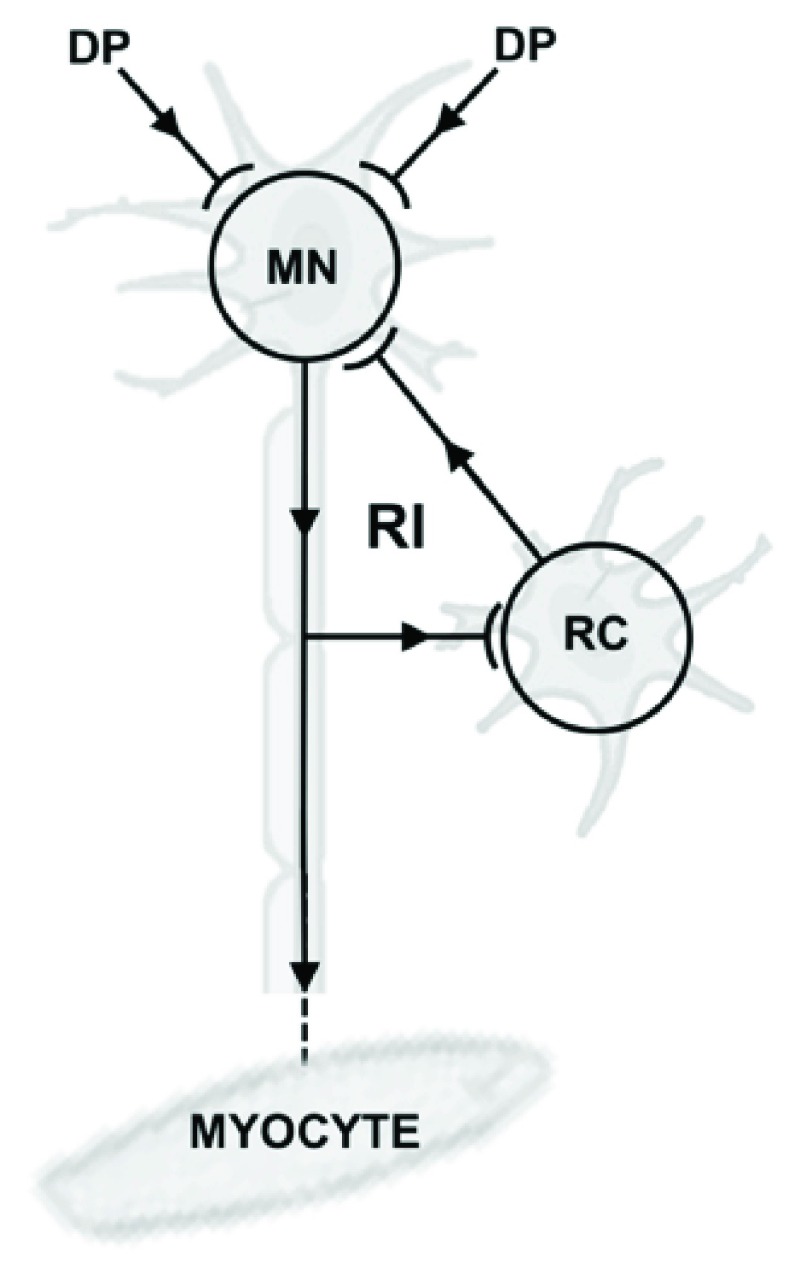
Schematic overview of recurrent inhibition. With recurrent inhibition (RI), input from descending pathways (DP) reaches the motor neuron (MN). In response, the MN activates the target myocyte; in addition, the MN also activates Renshaw cells (RC), which then inhibit the motor neuron through a negative feedback loop.

The inhibitory system functions via both SI and RI ^64^. The RI system controls physiological functions that play a role in counteracting gravitational forces and other external forces acting on the body. During locomotion and/or to counteract the effects of gravity, RI uses a negative inhibitory feedback loop ( Figure 1), thereby providing muscles with additional, stabilizing input. Therefore, muscles involved in movement and lifting heavy objects are subject to RI. Examples of RI-innervated muscles include the limb and thorax muscles, as well as the neck muscles that control head movement.

In contrast, neuronal pathways that do not play a role in locomotion or counteracting gravity selectively utilize SI ^64^. Examples of SI-innervated muscles include facial, speech, pharyngeal, and eye muscles, as well as muscles that are involved in bowel and bladder function.

Table 3 summarizes the involvement of SI and RI in the principal clinical manifestations of neuromuscular and/or neurological diseases. A close examination of Table 3 reveals that one set of muscles—namely, the respiratory muscles—is innervated by both SI and RI pathways ^64, 65^. This dual innervation arises because the respiratory muscles play a role in both respiratory function and maintaining body posture ^64^.

**Table 3.** Summary of neuronal pathways involved in neuromuscular and/or neurological diseases and their innervation by either simple inhibition (SI) or recurrent inhibition (RI) ^64^.

SI	RI	Neuronal pathways involved in neuromuscular and/or neurological diseases
√		Neuronal pathways controlling muscles involved in dysphagia
√		Neuronal pathways controlling muscles involved in dysarthria
√		Neuronal pathways controlling muscles involved in bowel function
√		Neuronal pathways controlling muscles involved in bladder function
√		Neuronal pathways controlling muscles involved in eye movement
√		Neuronal pathways controlling cognitive function
√		Neuronal pathways controlling respiratory muscles not involved in maintaining body posture
	√	Neuronal pathways controlling respiratory muscles involved in maintaining body posture
	√	Neuronal pathways controlling limb muscles involved in body locomotion or maintaining body posture

## SI and RI involvement can account for various onset manifestations in ALS

Strikingly, the categorization between SI-innervated and RI-innervated muscles coincides with the categorization of muscles affected in limb-onset ALS, bulbar-onset ALS, and respiratory-onset ALS. Approximately 70%, 25%, and 5% of ALS patients present initially with limb involvement (limb-onset ALS), bulbar symptoms (bulbar-onset ALS), or respiratory symptoms (respiratory-onset ALS), respectively ^2^, and this difference in onset can be explained by differences in SI versus RI involvement ( Table 4). Patients with both SI and RI involvement at the onset of disease present with respiratory-onset ALS. In contrast, patients with primarily SI involvement present with bulbar-onset ALS, whereas patients with primarily RI involvement present with limb-onset ALS (see Table 4). Thus, SI and RI differentiation can account for this difference in ALS onset.

**Table 4.** Limb-onset, bulbar-onset, and respiratory-onset ALS can be differentiated based on targets that are innervated by simple inhibition (SI) and/or recurrent inhibition (RI).

SI	RI	Projection target of affected neurons	ALS onset type
	√	Distal upper-limb muscles	Limb-onset ALS
	√	Proximal upper-limb muscles	
	√	Distal lower-limb muscles	
	√	Proximal lower-limb muscles	
	√	Respiratory muscles involved in maintaining body posture	Respiratory-onset ALS
√		Respiratory muscles not involved in maintaining body posture	
√		Speech muscles	Bulbar-onset ALS
√		Swallowing muscles	
√		Tongue, mouth, cheek, and palate muscles	
√		Bladder muscles	
√		Gastrointestinal muscles	
√		Eye muscles	
√		Facial movements	
√		Emotional function	
√		Cognitive function	

## SI versus RI involvement can account for differences in life expectancy among patients with ALS

The differential involvement of the SI or RI system can also explain the observed differences in life expectancy between patients who present with limb-onset, bulbar-onset, or respiratory-onset ALS. Specifically, patients with respiratory-onset ALS generally have the shortest life expectancy following diagnosis ^4^. As discussed above, increased activity of both the SI and RI pathways leads to fatal respiratory depression, the principal cause of early death in patients with ALS.

Increased activity of either the SI or RI pathway—but not both—can also lead to respiratory depression, albeit not to fatal levels. Under these conditions, respiratory function, though impaired, can be maintained by either SI or RI pathway activity. However, due to impaired respiratory function resulting from either SI or RI overstimulation (see Table 4), these patients can die from dysphagia-related malnutrition and/or aspiration pneumonia. Thus, patients with either SI or RI involvement—but not both—generally live longer than patients with both SI and RI involvement. Importantly, this observation can also explain why patients with motor neuron diseases at either end of the SI/RI spectrum—for example, primary lateral sclerosis, progressive muscular atrophy, progressive bulbar palsy, or pseudobulbar palsy—have a longer life expectancy than patients with ALS ^4^, which lies in the middle of the spectrum.

## The SI and RI pathways can explain both the progression of ALS into FTD and the progression from FTD into ALS

Differences between the effects of SI versus RI involvement can explain the fact that although ALS and FTD generally involve two distinct systems, these two diseases have a certain degree of overlap with respect to their clinical manifestations (see Table 1). Thus, if RI overstimulation precedes SI involvement, the patient can present with an initial diagnosis of ALS and can progress to ALS-FTD, a common manifestation of cognitive dysfunction observed in 20–50% of patients with late-state ALS patients ^2– 7^. Alternatively, if SI overstimulation precedes RI involvement, FTD is the initial diagnosis, and the disease can progress to FTD-ALS when the RI pathway becomes involved. Moreover, the division between SI and RI can also explain the overlap between subcategories of ALS and FTD with respect to impaired cognition and altered behavior that involve SI, and movement dysfunction that involves RI.

## Differential involvement of SI and RI can account for the wide variety of clinical manifestations in ALS

Although it is generally considered one disease, ALS can present with a wide spectrum of clinical manifestations, and this spectrum can be explained by the involvement of SI and/or RI pathways. For example, SI overstimulation can lead to bulbar, cognitive, and frontotemporal dementia-related manifestations without causing severe muscle wasting or respiratory malfunction (for example, as observed in patients with bulbar-onset ALS). On the other hand, RI overstimulation can lead to locked-in syndrome, a state in which the patient retains cognitive and emotional function but becomes “locked” in their body, with all of the muscles that counteract gravity and other external forces rendered essentially dysfunctional. Interestingly, the only muscles that are spared in locked-in syndrome—and the only way in which end-stage patients can communicate with the outside world—are the muscles that control eye movement. This is an important observation, given that the muscles that control eye movement are not controlled by RI pathways (see Table 3). The distinction between SI and RI can also explain the observation that some patients with ALS have fully intact cognitive and emotional functions even after their muscles involved in countering gravity have become dysfunctional; the most famous example of this phenomenon is Stephen Hawking, who despite being diagnosed with ALS in his early twenties remains active as a prominent theoretical physicist, now in his seventies.

## Split-hand syndrome in ALS can be explained by differential innervation of SI and RI pathways

Split-hand syndrome is common among patients with ALS ^66^. With split-hand syndrome, the abductor pollicis brevis (APB) and first dorsal interosseous (FDI) muscles are affected, whereas the abductor digiti minimi (ADM) muscle is relatively spared. This syndrome is particularly puzzling, as these muscles are innervated identically, yet are affected differently ^66^. I propose that split-hand syndrome can be attributed to differences in the extent to which RI pathways innervate the hand muscles that are involved in precision gripping, versus muscles that also play a role in power gripping. With precision gripping (for example, when using a pen), the fingers and thumb press against each other; this type of grip does not involve lifting a relatively heavy object ^67^. In contrast, power gripping (for example, when gripping a hammer or lifting a heavy pan) uses the fingers, palm, and thumb to clamp down on a heavy object in order to lift and control the object ^67^. Napier ^67^ used this distinction to distinguish muscle activities that are involved in body locomotion and/or posture from muscle activities that do not involve locomotion or posture. Thus, Napier’s separation also categorizes muscle activities into those that are controlled by RI and those that are controlled by SI. Because the primary function of the ADM muscle is to move the little finger (i.e., the fifth digit) away from the hand, the ADM muscle is only involved in precision gripping and would therefore not be affected by RI overstimulation. This is consistent with the reported absence of RI in motor neurons that innervate the ADM ^64, 68^. On the other hand, the APB and FDI muscles are involved in the opposition and extension of the thumb and are therefore involved in power gripping ^64^; thus, these two muscles are affected by RI overstimulation.

## Parkinson’s disease rest tremors can be attributed to differences between SI and RI involvement

In Parkinson’s disease, rest tremors arise from involuntary rhythmic oscillatory movements of a body part at rest; these tremors stop when the patient actively moves the affected body part. The pathways that underlie rest tremors have not been identified, and the fact that rest tremors resolve during voluntary movement is one of the most puzzling observations associated with Parkinson’s disease ^69^. However, because these tremors occur at rest (and not during active motion or while countering the effects of gravity), the muscles involved are likely innervated by SI pathways, making rest tremors an SI-specific phenomenon. This is further illustrated by the finding that rest tremors resolve when the affected body part becomes involved in locomotion, stance, or maintaining inertia ^69^, actions that involve muscles that are controlled by RI ^64^. Interestingly, the hand tremor that is most specific to patients with Parkinson’s disease—the so-called “pill-rolling tremor”—also results from muscles that are innervated solely by SI pathways. Specifically, the pill-rolling tremor involves muscles that play a role in precision gripping but not in power gripping, a distinction that is highly reminiscent of split-hand syndrome in ALS (see above). Furthermore, involvement of the inhibitory system in Parkinson’s disease rest tremors is supported by the observation that the rest tremors observed in restless legs syndrome resolve after the administration of quinine (FDA Drug safety communication, 2010), a compound that reduces inhibitory activity ^70^.

## The differentiation of clinical manifestations in neuromuscular and neurological diseases can be attributed to SI versus RI pathways

The differentiation between SI and RI summarized in Table 3 can explain the three categories of fatal symptoms that arise in end-stage neuromuscular and neurological diseases ( Table 5). One striking observation from Table 5 is that both SI and RI can be attributed to fatal respiratory failure, the major cause of death among ALS patients. Overstimulation of SI pathways leads to bowel dysfunction, bladder dysfunction, and dysphagia-related malnutrition and aspiration pneumonia; these symptoms are the major causes of death among patients with FTD, Alzheimer’s disease, Parkinson’s disease, and Huntington’s disease. On the other hand, overstimulation of RI pathways can lead to end-stage locked-in syndrome.

**Table 5.** The fatal symptom categories associated with neuromuscular and neurological diseases can be attributed to simple inhibition (SI) and/or recurrent inhibition (RI).

SI	RI	Fatal symptoms	Locked-in syndrome	ALS	FTD	Alzheimer’s disease	Parkinson’s disease	Huntington’s disease
√		Dysphagia-related malnutrition		√	√	√	√	√
√		Dysphagia-related aspiration pneumonia		√	√	√	√	√
√		Bowel dysfunction		√	√	√	√	√
√		Bladder dysfunction		√	√	√	√	√
√	√	Respiratory malfunction		√				
	√	Complete dysfunction of muscles involved in countering gravity	√	√				

Differential SI and RI involvement can also account for the wide variety of clinical manifestations in neuromuscular and neurological diseases during disease progression. As a group, neuromuscular and neurological diseases present with a wide spectrum of clinical manifestations (see Table 1), and stimulation of SI and/or RI pathways can account for this spectrum. For example, SI overstimulation can lead to FTD, Alzheimer’s disease, Parkinson’s disease, and Huntington’s disease, whereas RI overstimulation can lead to locked-in syndrome. Finally, overstimulation of both the SI and RI pathways can lead to ALS.

Finally, the differentiation between SI and RI can help explain the differences in life expectancy among patients with various neuromuscular and neurological diseases. As discussed above, increased activity of both the SI and RI pathways leads to fatal respiratory depression (see Table 5), the principal cause of death in patients with ALS, the neuromuscular disease with the shortest life expectancy. Increased activity of either the SI or RI pathway—but not both—can also lead to respiratory depression, albeit not to direct fatal levels. Thus, patients with either SI or RI overstimulation generally live longer than patients with both SI and RI overstimulation. This coincides with the observation that patients with ALS—in which both the SI and RI pathways are overstimulated—have a shorter life expectancy than patients with FTD, Alzheimer’s disease, Parkinson’s disease, Huntington’s disease, and locked-in syndrome, diseases in which either SI or RI activity is increased.

## Homeostatic interactions between inhibitory transmission and excitatory transmission

**Figure 2.**
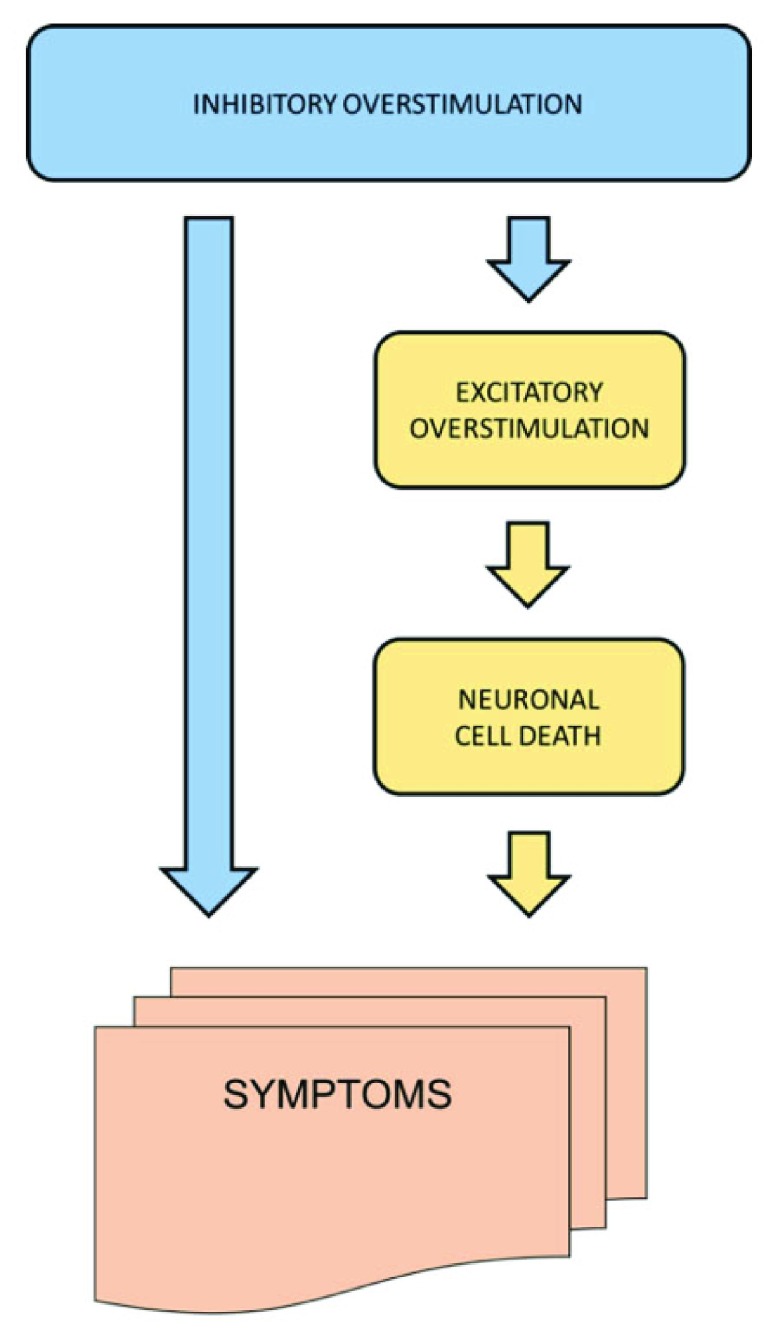
Schematic overview of glutamatergic overstimulation (yellow) and the inhibitory overstimulation hypothesis (blue and yellow). In the inhibitory overstimulation hypothesis, excitatory overstimulation is a homeostatic response to inhibitory overstimulation. A key feature of this model is that inhibitory overstimulation can be sufficient to cause symptoms (left blue arrow). As the disease progresses, increasing inhibitory overstimulation can eventually lead to excitatory overstimulation and neuronal cell death, making the symptoms irreversible.

Taken together, the wealth of observations discussed above suggest that the opposing excitatory and inhibitory systems may play a role in the pathogenesis of the same disease. This phenomenon has precedent, as inhibitory/excitatory homeostasis processes are also involved in seizure activity ^30, 31^. Neurons that receive excessive excitatory stimulation can subsequently become overstimulated by inhibitory transmission, and vice versa. This raises the intriguing question of which system in neuromuscular and neurological diseases is overstimulated first, and which system becomes overstimulated as a homeostatic response. This question has been addressed with respect to epileptic seizures ^30^. With respect to neuromuscular and neurological diseases, it is important to note that the administration of glutamatergic excitatory compounds does not lead to the clinical manifestations summarized in Table 1; glutamatergic overstimulation can give rise to clinical manifestations only through excitotoxicity (i.e., overstimulation-induced neuronal cell death). However, inhibitory overstimulation *can* give rise to the clinical manifestations in Table 1, even in the absence of neuronal cell death. Thus, I hypothesize that inhibitory overstimulation occurs first, and excitatory overstimulation is a homeostatic response. As inhibitory overstimulation increases, the excitatory system is stimulated further, until it reaches a level that induces neuronal cell death. This process is depicted schematically in Figure 2. Importantly, the order of the homeostatic process hypothesized here is precisely opposite to the homeostatic processes observed during epileptic seizures, in which excitatory overstimulation proceeds inhibitory overstimulation ^30^.

## Other possible interpretations of these observations

Other possible interpretations of the findings summarized in Table 1 should be considered. If inhibitory overstimulation plays a key role in neuromuscular and neurological diseases, one would expect these patients to present with sedation, thus indicating the possibility that physiological systems other than the inhibitory system may be involved. However, not all GABA receptor subtypes are involved in sedation ^71, 72^. Thus, inhibitory activity can occur without inducing pronounced sedation. This is supported by reports that benzodiazepine-induced dysphagia can occur even in non-sedated patients ^40– 42^. Second, the absence of seizures despite high glutamate levels could be due to a slow, but non-epileptogenic increase in glutamate levels during the progression of neuromuscular and neurological diseases. However, even small increases in glutamate levels can increase glutamatergic synchronization of a small subset of critical neurons, thereby leading to epileptic activity ^30, 31^. Moreover, epileptic seizures are simply not reported among ALS patients, an observation that cannot be explained by a slow increase in glutamate levels, as glutamate does not cause inhibitory activity ^22– 29^. Furthermore, slow increasing levels of glutamate in the absence of seizure activity may reflect the involvement of an inhibitory homeostatic process ^30, 31^. Finally, the differentiation between SI and RI depicted in Table 4 and Table 5 may be attributed to the involvement of neuronal pathways projecting to either voluntary or involuntary muscles. However, this cannot explain split-hand syndrome in ALS patients or rest tremors in Parkinson’s disease, as these phenomena involve only voluntary muscles. Moreover, split-hand syndrome involves identically innervated muscles that cannot be differentiated by any aspect other than SI/RI innervation.

## Therapeutic potential for targeting inhibitory activity

From a clinical perspective, an important consequence that emerges from the inhibitory overstimulation hypothesis is that the clinical manifestations summarized in Table 1 develop *before* neurons have undergone cell death. The implication of this possibility is that decreasing inhibitory activity may be beneficial in terms of slowing—or even preventing—the progression of neuromuscular and neurological diseases. Compounds that can reduce inhibitory activity are currently available; unfortunately, however, these compounds can induce seizure activity and are therefore not used therapeutically. Nevertheless, their potential for preventing the pathogenesis of neuromuscular and neurological diseases suggests that compounds that target the inhibitory system could be developed for clinical applications. For example, the average life expectancy of a patient with ALS is 3–5 years after onset, and most neuromuscular and neurological diseases are severe and ultimately fatal. With respect to Alzheimer’s disease and Parkinson’s disease, dysphagia and respiratory depression–related aspiration pneumonia are the most common causes of death ^16, 17^. Neuromuscular diseases also present with the severe and potential fatal clinical manifestations listed in Table 1; ^1– 7, 9– 11, 13, 15, 17– 21^ thus, the ability to prevent these symptoms could significantly prolong the life expectancy of these patients. From a treatment perspective, it is interesting to note that the selective GABA antagonist SGS-742 has been shown to be both clinically feasible and safe ^55^.

## Conclusions and future perspectives

Based upon the plethora of observations regarding the inhibitory system, I hypothesize that this system plays an important role in the pathogenesis of both neuromuscular and neurological diseases. Importantly, overstimulation of the inhibitory system can explain both the absence of epileptic seizures despite the elevated glutamate levels and the pharmacological induction of symptoms present in patients with neuromuscular and neurological diseases. Moreover, the separation between SI and RI can account for the various categories of clinical manifestations observed in these patients. Specifically, I hypothesize that increased glutamate levels in neuromuscular and neurological diseases are actually a homeostatic response to an overstimulated inhibitory system. Implicating the inhibitory system in the pathogenesis of neuromuscular and neurological diseases is highly relevant, given that the majority of approaches being developed for treating these diseases focus on reducing glutamatergic activity, rather than reducing inhibitory activity. Moreover, this putative connection between the inhibitory system and neuromuscular/neurological diseases may have long-reaching implications, including the need to develop therapies designed to reduce inhibitory overstimulation in neuromuscular and neurological patients.

## References

[1] Standifer: Handbook of disabilities, neuromuscular disorders, RCEP7, University of Missouri,2014.

[2] Kiernan MC Vucic S Cheah BC: Amyotrophic lateral sclerosis. *Lancet.* 2011;377(9769):942–55. 10.1016/S0140-6736(10)61156-7 21296405

[3] Silani V Messina S Poletti B: The diagnosis of Amyotrophic lateral sclerosis in 2010. *Arch Ital Biol.* 2011;149(1):5–27. 10.4449/aib.v149i1.1260 21412713

[4] Turner MR Hardiman O Benatar M: Controversies and priorities in amyotrophic lateral sclerosis. *Lancet Neurol.* 2013;12(3):310–22. 10.1016/S1474-4422(13)70036-X 23415570 PMC4565161

[5] Achi EY Rudnicki SA: ALS and Frontotemporal Dysfunction: A Review. *Neurol Res Int.* 2012;2012: 806306. 10.1155/2012/806306 22919484 PMC3423946

[6] Zago S Poletti B Morelli C: Amyotrophic lateral sclerosis and frontotemporal dementia (ALS-FTD). *Arch Ital Biol.* 2011;149(1):39–56. 10.4449/aib.v149i1.1263 21412715

[7] Ringholz GM Appel SH Bradshaw M: Prevalence and patterns of cognitive impairment in sporadic ALS. *Neurology.* 2005;65(4):586–90. 10.1212/01.wnl.0000172911.39167.b6 16116120

[8] Warren JD Rohrer JD Rossor MN: Clinical review. Frontotemporal dementia. *BMJ.* 2013;347:f4827. 10.1136/bmj.f4827 23920254 PMC3735339

[9] Tartaglia MC Rowe A Findlater K: Differentiation between primary lateral sclerosis and amyotrophic lateral sclerosis: examination of symptoms and signs at disease onset and during follow-up. *Arch Neurol.* 2007;64(2):232–6. 10.1001/archneur.64.2.232 17296839

[10] Singer MA Statland JM Wolfe GI: Primary lateral sclerosis. *Muscle Nerve.* 2007;35(3):291–302. 10.1002/mus.20728 17212349

[11] Kalia LV Lang AE: Parkinson's disease. *Lancet.* 2015;386(9996):896–912. 10.1016/S0140-6736(14)61393-3 25904081

[12] Alves L Correia AS Miguel R: Alzheimer’s disease: a clinical practice-oriented review. *Front Neurol.* 2012;3: 63. 10.3389/fneur.2012.00063 22529838 PMC3330267

[13] Roos RA: Huntington's disease: a clinical review. *Orphanet J Rare Dis.* 2010;5:40. 10.1186/1750-1172-5-40 21171977 PMC3022767

[14] Spencer D: Seizures and epileptiform activity in early Alzheimer disease: how hard should we be looking? *Epilepsy Curr.* 2014;14(2):73–75. 10.5698/1535-7597-14.2.73 24872782 PMC4010880

[15] Martino D Stamelou M Bhatia KP: The differential diagnosis of Huntington's disease-like syndromes: 'red flags' for the clinician. *J Neurol Neurosurg Psychiatry.* 2013;84(6):650–656. 10.1136/jnnp-2012-302532 22993450 PMC3646286

[16] Kalia M: Dysphagia and aspiration pneumonia in patients with Alzheimer's disease. *Metabolism.* 2003;52(10 Suppl 2):36–8. 10.1016/S0026-0495(03)00300-7 14577062

[17] Tjaden K: Speech and Swallowing in Parkinson’s Disease. *Top Geriatr Rehabil.* 2008;24(2):115–126. 10.1097/01.TGR.0000318899.87690.44 19946386 PMC2784698

[18] Cohen B Caroscio J: Eye movements in amyotrophic lateral sclerosis. *J Neural Transm Suppl.* 1983;19:305–15. 6583314

[19] Panicker JN Fowler CJ Kessler TM: Lower urinary tract dysfunction in the neurological patient: clinical assessment and management. *Lancet Neurol.* 2015;14(7):720–32. 10.1016/S1474-4422(15)00070-8 26067125

[20] Toepfer M Folwaczny C Klauser A: Gastrointestinal dysfunction in amyotrophic lateral sclerosis. *Amyotroph Lateral Scler Other Motor Neuron Disord.* 1999;1(1):15–9. 10.1080/146608299300079484 12365061

[21] Fasano A Visanji NP Liu LW: Gastrointestinal dysfunction in Parkinson's disease. *Lancet Neurol.* 2015;14(6):625–39. 10.1016/S1474-4422(15)00007-1 25987282

[22] Rothstein JD Tsai G Kuncl RW: Abnormal excitatory amino acid metabolism in amyotrophic lateral sclerosis. *Ann Neurol.* 1990;28(1):18–25. 10.1002/ana.410280106 2375630

[23] Spreux-Varoquaux O Bensimon G Lacomblez L: Glutamate levels in cerebrospinal fluid in amyotrophic lateral sclerosis: a reappraisal using a new HPLC method with coulometric detection in a large cohort of patients. *J Neurol Sci.* 2002;193(2):73–78. 10.1016/S0022-510X(01)00661-X 11790386

[24] Olney JW: Neurotoxicity of excitatory amino acids. In: E.G. McGeer, J.W. Olney, P.L. McGeer (Eds.), Kainic Acid as a Tool in Neurobiology, Raven Press, New York,1978;95–121.

[25] Coyle JT Puttfarcken P: Oxidative stress, glutamate, and neurodegenerative disorders. *Science.* 1993;262(5134):689–695. 10.1126/science.7901908 7901908

[26] Kim AH Kerchner GA Choi DW: Blocking Excitotoxicity or Glutamatergic Storm.Chapter 1 in CNS Neuroprotection. Marcoux FW and Choi DW, editors. Springer, New York.2002;3–36. Reference Source

[27] Lipton SA Rosenberg PA: Excitatory amino acids as a final common pathway for neurologic disorders. *N Engl J Med.* 1994;330(9):613–622. 10.1056/NEJM199403033300907 7905600

[28] Doble A: The role of excitotoxicity in neurodegenerative disease: implications for therapy. *Pharmacol Ther.* 1999;81(3):163–221. 10.1016/S0163-7258(98)00042-4 10334661

[29] Dong XX Wang Y Qin ZH: Molecular mechanisms of excitotoxicity and their relevance to pathogenesis of neurodegenerative diseases. *Acta Pharmacol Sin.* 2009;30(4):379–87. 10.1038/aps.2009.24 19343058 PMC4002277

[30] During MJ Spencer DD: Extracellular hippocampal glutamate and spontaneous seizure in the conscious human brain. *Lancet.* 1993;341(8861):1607–10. 10.1016/0140-6736(93)90754-5 8099987

[31] Chamberlin NL Traub RD Dingledine R: Role of EPSPs in initiation of spontaneous synchronized burst firing in rat hippocampal neurons bathed in high potassium. *J Neurophysiol.* 1990;64(3):1000–8. 1977893 10.1152/jn.1990.64.3.1000

[32] Scarmeas N Honig LS Choi H: Seizures in Alzheimer disease: who, when, and how common? *Arch Neurol.* 2009;66(8):992–7. 10.1001/archneurol.2009.130 19667221 PMC2768279

[33] Amatniek JC Hauser WA DelCastillo-Castaneda C: Incidence and predictors of seizures in patients with Alzheimer's disease. *Epilepsia.* 2006;47(5):867–72. 10.1111/j.1528-1167.2006.00554.x 16686651

[34] Möhler H Crestani F Rudolph U: GABA _A_-receptor subtypes: a new pharmacology. *Curr Opin Pharmacol.* 2001;1(1):22–5. 10.1016/S1471-4892(01)00008-X 11712530

[35] Rudolph U Knoflach F: Beyond classical benzodiazepines: Novel therapeutic potential of GABA _A_ receptor subtypes. *Nat Rev Drug Discov.* 2011;10(9):685–697. 10.1038/nrd3502 21799515 PMC3375401

[36] Forster A Gardaz JP Suter PM: Respiratory depression by midazolam and diazepam. *Anesthesiology.* 1980;53(6):494–7. 10.1097/00000542-198012000-00010 7457966

[37] Wall BT Dirks ML Snijders T: Substantial skeletal muscle loss occurs during only 5 days of disuse. *Acta Physiol (Oxf).* 2014;210(3):600–11. 10.1111/apha.12190 24168489

[38] Sullivan EV Pfefferbaum A: Alcohol and the Nervous System: Handbook of Clinical Neurology.Edited Edith V. Sullivan & Adolf Pfefferbaum. ISBN9780444626226.2014;95–96.

[39] Hockman CH Weerasuriya A Bieger D: GABA receptor-mediated inhibition of reflex deglutition in the cat. *Dysphagia.* 1996;11(3):209–215. 10.1007/BF00366388 8755468

[40] Wyllie E Wyllie R Cruse RP: The mechanism of nitrazepam-induced drooling and aspiration. *N Engl J Med.* 1986;314(1):35–8. 10.1056/NEJM198601023140107 3940315

[41] Buchholz D Jones B Neumann S: Benzodiazepine-induced pharyngeal dysphagia: a report of two probable cases. *Dysphagia.* 1995;10(2):142–6.

[42] Dantas RO Nobre Souza MA: Dysphagia induced by chronic ingestion of benzodiazepine. *Am J Gastroenterol.* 1997;92(7):1194–6. 9219798

[43] Dua KS Surapaneni SN Santharam R: Effect of systemic alcohol and nicotine on airway protective reflexes. *Am J Gastroenterol.* 2009;104(10):2431–8. 10.1038/ajg.2009.330 19550414 PMC4160881

[44] Van Steveninck AL Mandema JW Tuk B: A comparison of the concentration-effect relationships of midazolam for EEG-derived parameters and saccadic peak velocity. *Br J Clin Pharmacol.* 1993;36(2):109–115. 10.1111/j.1365-2125.1993.tb04205.x 8398578 PMC1364573

[45] Benson PJ Beedie SA Shephard E: Simple viewing tests can detect eye movement abnormalities that distinguish schizophrenia cases from controls with exceptional accuracy. *Biol Psychiatry.* 2012;72(9):716–24. 10.1016/j.biopsych.2012.04.019 22621999

[46] Iyer PM Egan C Pinto-Grau M: Functional Connectivity Changes in Resting-State EEG as Potential Biomarker for Amyotrophic Lateral Sclerosis. *PLoS One.* 2015;10(6):e0128682. 10.1371/journal.pone.0128682 26091258 PMC4474889

[47] Igawa Y Mattiasson A Andersson KE: Effects of GABA-receptor stimulation and blockade on micturition in normal rats and rats with bladder outflow obstruction. *J Urol.* 1993;150(2 Pt 1):537–42. 8392118 10.1016/s0022-5347(17)35542-8

[48] Andersson KE Wein AJ: Pharmacology of the lower urinary tract: basis for current and future treatments of urinary incontinence. *Pharmacol Rev.* 2004;56(4):581–631. 10.1124/pr.56.4.4 15602011

[49] Hyland NP Cryan JF: A Gut Feeling about GABA: Focus on GABA _B_ Receptors. *Front Pharmacol.* 2010;1:124. 10.3389/fphar.2010.00124 21833169 PMC3153004

[50] Krantis A: GABA in the Mammalian Enteric Nervous System. *News Physiol Sci.* 2000;15:284–290. 11390928 10.1152/physiologyonline.2000.15.6.284

[51] Bravo JA Forsythe P Chew MV: Ingestion of *Lactobacillus* strain regulates emotional behavior and central GABA receptor expression in a mouse via the vagus nerve. *Proc Natl Acad Sci U S A.* 2011;108(38):16050–5. 10.1073/pnas.1102999108 21876150 PMC3179073

[52] Oslin D Atkinson RM Smith DM: Alcohol related dementia: proposed clinical criteria. *Int J Geriatr Psychiatry.* 1998;13(4):203–212. 10.1002/(SICI)1099-1166(199804)13:4<203::AID-GPS734>3.0.CO;2-B 9646147

[53] Billioti de Gage S Bégaud B Bazin F: Benzodiazepine use and risk of dementia: prospective population based study. *BMJ.* 2012;345(2012):e6231. 10.1136/bmj.e6231 23045258 PMC3460255

[54] Bell GD Spickett GP Reeve PA: Intravenous midazolam for upper gastrointestinal endoscopy: a study of 800 consecutive cases relating dose to age and sex of patient. *Br J Clin Pharmacol.* 1987;23(2):241–243. 10.1111/j.1365-2125.1987.tb03037.x 3828200 PMC1386076

[55] Froestl W Gallagher M Jenkins H: SGS742: the first GABA _B_ receptor antagonist in clinical trials. *Biochem Pharmacol.* 2004;68(8):1479–87. 10.1016/j.bcp.2004.07.030 15451390

[56] Martínez-Cué C Delatour B Potier MC: Treating enhanced GABAergic inhibition in Down syndrome: Use of GABA α5-selective inverse agonists. *Neurosci Biobehav Rev.* 2014;46(Pt 2):218–227. 10.1016/j.neubiorev.2013.12.008 24412222

[57] Wu Z Guo Z Gearing M: Tonic inhibition in dentate gyrus impairs long-term potentiation and memory in an Alzheimer's [corrected] disease model. *Nat Commun.* 2014;5:4159. 10.1038/ncomms5159 24923909 PMC4159602

[58] Miller RG Moore DH 2nd Gelinas DF: Phase III randomized trial of gabapentin in patients with amyotrophic lateral sclerosis. *Neurology.* 2001;56(7):843–848. 11294919 10.1212/wnl.56.7.843

[59] Cudkowicz ME Shefner JM Schoenfeld DA: A randomized, placebo-controlled trial of topiramate in amyotrophic lateral sclerosis. *Neurology.* 2003;61(4):456–464. 10.1212/WNL.61.4.456 12939417

[60] Gordon PH Moore DH Miller RG: Efficacy of minocycline in patients with amyotrophic lateral sclerosis: a phase III randomised trial. *Lancet Neurol.* 2007;6(12):1045–53. 10.1016/S1474-4422(07)70270-3 17980667

[61] Ahmadirad N Shojaei A Javan M: Effect of minocycline on pentylenetetrazol-induced chemical kindled seizures in mice. *Neurol Sci.* 2014;35(4):571–6. 10.1007/s10072-013-1552-0 24122023

[62] Elia AE Lalli S Monsurrò MR: Tauroursodeoxycholic acid in the treatment of patients with amyotrophic lateral sclerosis. *Eur J Neurol.* 2016;23(1):45–52. 10.1111/ene.12664 25664595 PMC5024041

[63] Yanovsky Y Schubring SR Yao Q: Waking action of ursodeoxycholic acid (UDCA) involves histamine and GABA _A_ receptor block. *PLoS One.* 2012;7(8):e42512. 10.1371/journal.pone.0042512 22880010 PMC3412845

[64] Windhorst U: On the role of recurrent inhibitory feedback in motor control. *Prog Neurobiol.* 1996;49(6):517–87. 10.1016/0301-0082(96)00023-8 8912393

[65] Lipski J Fyffe RE Jodkowski J: Recurrent inhibition of cat phrenic motoneurons. *J Neurosci.* 1985;5(6):1545–55. 4009244 10.1523/JNEUROSCI.05-06-01545.1985 PMC6565266

[66] Eisen A Kuwabara S: The split hand syndrome in amyotrophic lateral sclerosis. *J Neurol Neurosurg Psychiatry.* 2012;83(4):399–403. 10.1136/jnnp-2011-301456 22100761

[67] Napier JR: The prehensile movements of the human hand. *J Bone Joint Surg Br.* 1956;38-B(4):902–913. 13376678 10.1302/0301-620X.38B4.902

[68] Rossi A Mazzocchio R: Renshaw recurrent inhibition to motoneurones innervating proximal and distal muscles of the human upper and lower limbs.In: Muscle Afferents and Spinal Control of Movement, Eds L. Jami, E. Pierrot-DeseilIigny and D Zytnicki. Pergamon Press: Oxford.1992;313–319.

[69] Helmich RC Hallett M Deuschl G: Cerebral causes and consequences of parkinsonian resting tremor: a tale of two circuits? *Brain.* 2012;135(Pt 11):3206–26. 10.1093/brain/aws023 22382359 PMC3501966

[70] Amabeoku GJ Chikuni O: Effects of some GABAergic agents on quinine-induced seizures in mice. *Experientia.* 1992;48(7):659–62. 10.1007/BF02118313 1322322

[71] McKernan RM Rosahl TW Reynolds DS: Sedative but not anxiolytic properties of benzodiazepines are mediated by the GABA(A) receptor alpha1 subtype. *Nat Neurosci.* 2000;3(6):587–92. 10.1038/75761 10816315

[72] Reynolds DS Rosahl TW Cirone J: Sedation and anesthesia mediated by distinct GABA(A) receptor isoforms. *J Neurosci.* 2003;23(24):8608–17. 13679430 10.1523/JNEUROSCI.23-24-08608.2003 PMC6740367

