# Supplementary material for: Overstimulation of the inhibitory nervous system plays a role in the pathogenesis of neuromuscular and neurological diseases: a novel hypothesis
Source: F1000Res. 2016 Aug 19;5:1435. Originally published 2016 Jun 20. [Version 2] doi: 10.12688/f1000research.8774.2 (PMC4984481; doi:10.12688/f1000research.8774.2)
Supplement: Peer review history

# Peer review: Referee response for version 2

**DOI:** 10.5256/f1000research.10177.r15790

**Published:** 2016-09-23

**Authors:** Willem M de Vos

**Affiliations:** 1 Laboratory of Microbiology, Wageningen University, Wageningen, Netherlands

I have read this submission. I believe that I have an appropriate level of expertise to confirm that it is of an acceptable scientific standard.

# Peer review: Referee response for version 2

**DOI:** 10.5256/f1000research.10177.r15968

**Published:** 2016-08-31

**Authors:** Janet Hoogstraate

**Affiliations:** 1 Valneva Sweden AB, Stockholm, Sweden; 2 Stockholm Brain Institute, Stockholm, Sweden

I have read this submission. I believe that I have an appropriate level of expertise to confirm that it is of an acceptable scientific standard.

# Peer review: Referee response for version 1

**DOI:** 10.5256/f1000research.9443.r15213

**Published:** 2016-08-10

**Authors:** Willem M de Vos

**Affiliations:** 1 Laboratory of Microbiology, Wageningen University, Wageningen, Netherlands

The opinion paper by Tuk provides an overview of the inhibitory system involved in neural signal transduction. Based on an extensive review of the literature the author provides a number of challenging but well-reasoned hypotheses about the impact of this system on both neuromuscular and neurological diseases. This potential connection may have important implications for the understanding and future treatment of these so far (almost) untreatable diseases.

 Only few minor points should be addressed.  

  

1. The use of the term ‘inhibitory system’ is somewhat confusing – the context is often not specified (inhibitory system of what…) although the author means neural signal transduction – however,  this should be mentioned where relevant and certainly in the title.
2. The link between GABA production by intestinal bacteria and gut GABA receptor stimulation could be further addressed as it provides a way how other factors such as food or the gut microbiome may modulate GABA response (see also reference 47 and later work of these authors like Bravo *et al. *(2011).
3. The literature is well reviewed and the paper is supported by 5 useful Tables, 2 clear Figures and 70 references of peer reviewed papers – the one Washington Post article may be better mentioned in the text.

I have read this submission. I believe that I have an appropriate level of expertise to confirm that it is of an acceptable scientific standard.

## References

[1] BravoJAForsythePChewMVEscaravageESavignacHMDinanTGBienenstockJCryanJF: Ingestion of Lactobacillus strain regulates emotional behavior and central GABA receptor expression in a mouse via the vagus nerve. *Proc Natl Acad Sci U S A*.2011;108(38) : 10.1073/pnas.1102999108 16050-5 10.1073/pnas.1102999108 21876150PMC3179073

# Peer review: Referee response for version 1

**DOI:** 10.5256/f1000research.9443.r15214

**Published:** 2016-08-05

**Authors:** Janet Hoogstraate

**Affiliations:** 1 Valneva Sweden AB, Stockholm, Sweden; 2 Stockholm Brain Institute, Stockholm, Sweden

The manuscript by B.Tuk provides a comprehensive and up-to-date review of the clinical observations of neurodegenerative diseases relevant to the discussion on pathogenesis. The methodology of linking the clinical observations to a common pathology that is different in sequence of events compared to other scientific literature is sound. The data and the proposed theory are presented in a suitable manner.

 The position that there is an absence of elevated seizure activity in all 8 of the neurological and neuromuscular diseases should be revised to my opinion. While this can be said for ALS, there is substantial epidemiological data to support an higher frequency of epileptic seizures in the early stages of Alzheimer's Disease (ref 1-5). Based on the enormous increase of glutamate levels in ALS patients and the absence of elevated risk for epileptic activity in that population, there is still good support for postulating the theory of the inhibitory system as a plausible trigger for these diseases, but I propose that that section of the article and Table 1 be revised to reflect that nuance.

 I recommend that the article be indexed, with the minor revision outlined above.

I have read this submission. I believe that I have an appropriate level of expertise to confirm that it is of an acceptable scientific standard.

## References

[1] ImfeldPBodmerMSchuerchMJickSSMeierCR: Seizures in patients with Alzheimer's disease or vascular dementia: a population-based nested case-control analysis. *Epilepsia*.2013;54(4) : 10.1111/epi.12045 700-7 10.1111/epi.12045 23215680

[2] GaitatzisACarrollKMajeedAW SanderJ: The epidemiology of the comorbidity of epilepsy in the general population. *Epilepsia*.2004;45(12) : 10.1111/j.0013-9580.2004.17504.x 1613-22 10.1111/j.0013-9580.2004.17504.x 15571520

[3] FriedmanDHonigLSScarmeasN: Seizures and epilepsy in Alzheimer's disease. *CNS Neurosci Ther*.2012;18(4) : 10.1111/j.1755-5949.2011.00251.x 285-94 10.1111/j.1755-5949.2011.00251.x 22070283PMC3630499

[4] ScarmeasNHonigLSChoiHCanteroJBrandtJBlackerDAlbertMAmatniekJCMarderKBellKHauserWASternY: Seizures in Alzheimer disease: who, when, and how common?. *Arch Neurol*.2009;66(8) : 10.1001/archneurol.2009.130 992-7 10.1001/archneurol.2009.130 19667221PMC2768279

[5] AmatniekJCHauserWADelCastillo-CastanedaCJacobsDMMarderKBellKAlbertMBrandtJSternY: Incidence and predictors of seizures in patients with Alzheimer's disease. *Epilepsia*.2006;47(5) : 10.1111/j.1528-1167.2006.00554.x 867-72 10.1111/j.1528-1167.2006.00554.x 16686651
